# Automatic Artery/Vein Classification Using a Vessel-Constraint Network for Multicenter Fundus Images

**DOI:** 10.3389/fcell.2021.659941

**Published:** 2021-06-11

**Authors:** Jingfei Hu, Hua Wang, Zhaohui Cao, Guang Wu, Jost B. Jonas, Ya Xing Wang, Jicong Zhang

**Affiliations:** ^1^School of Biological Science and Medical Engineering, Beihang University, Beijing, China; ^2^Hefei Innovation Research Institute, Beihang University, Hefei, China; ^3^Beijing Advanced Innovation Centre for Biomedical Engineering, Beihang University, Beijing, China; ^4^School of Biomedical Engineering, Anhui Medical University, Hefei, China; ^5^Beijing Institute of Ophthalmology, Beijing Tongren Hospital, Capital Medical University, Beijing Ophthalmology and Visual Sciences Key Laboratory, Beijing, China; ^6^Department of Ophthalmology, Medical Faculty Mannheim of the Ruprecht-Karls-University Heidelberg, Mannheim, Germany; ^7^Beijing Advanced Innovation Centre for Big Data-Based Precision Medicine, Beihang University, Beijing, China

**Keywords:** vessel constraint, artery/vein classification, vessel segmentation, multi-center, data fusion

## Abstract

Retinal blood vessel morphological abnormalities are generally associated with cardiovascular, cerebrovascular, and systemic diseases, automatic artery/vein (A/V) classification is particularly important for medical image analysis and clinical decision making. However, the current method still has some limitations in A/V classification, especially the blood vessel edge and end error problems caused by the single scale and the blurred boundary of the A/V. To alleviate these problems, in this work, we propose a vessel-constraint network (VC-Net) that utilizes the information of vessel distribution and edge to enhance A/V classification, which is a high-precision A/V classification model based on data fusion. Particularly, the VC-Net introduces a vessel-constraint (VC) module that combines local and global vessel information to generate a weight map to constrain the A/V features, which suppresses the background-prone features and enhances the edge and end features of blood vessels. In addition, the VC-Net employs a multiscale feature (MSF) module to extract blood vessel information with different scales to improve the feature extraction capability and robustness of the model. And the VC-Net can get vessel segmentation results simultaneously. The proposed method is tested on publicly available fundus image datasets with different scales, namely, DRIVE, LES, and HRF, and validated on two newly created multicenter datasets: Tongren and Kailuan. We achieve a balance accuracy of 0.9554 and F1 scores of 0.7616 and 0.7971 for the arteries and veins, respectively, on the DRIVE dataset. The experimental results prove that the proposed model achieves competitive performance in A/V classification and vessel segmentation tasks compared with state-of-the-art methods. Finally, we test the Kailuan dataset with other trained fusion datasets, the results also show good robustness. To promote research in this area, the Tongren dataset and source code will be made publicly available. The dataset and code will be made available at https://github.com/huawang123/VC-Net.

## Introduction

**FIGURE 1:**
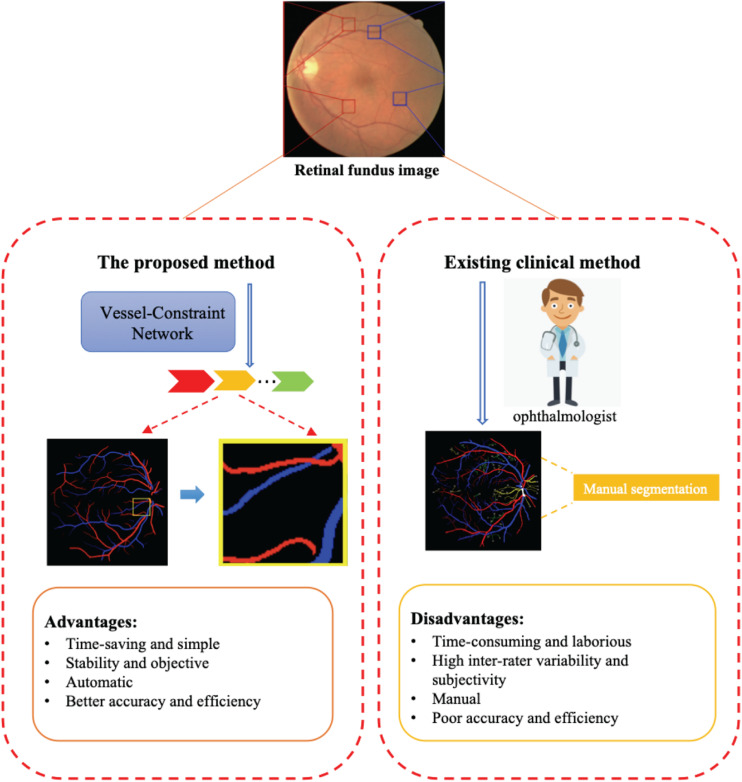
The proposed method can automatically and efficiently classify artery/vein (A/V) and segmented vessels from a retinal fundus image. The advantages of this method are its great help to ophthalmologists compared with existing clinical methods.

Retinal blood vessels have attracted widespread research efforts as these vessels represent the only internal human vascular structures that can be observed noninvasively. Retinal vessel abnormalities reflect the cumulative damage caused by chronic diseases such diabetes and hypertension and represent an important risk indicator for many systemic and cardiovascular diseases (Wong et al., 2004). And the artery/vein (A/V) may be affected differently by variations in disease types and progression. For example, artery narrowing is mostly associated with arterial hypertension, whereas vein widening is related to increased brain pressure, stroke, and similar cardiovascular diseases. Hence, accurate image-based analysis and evaluation methods for the morphological evaluation of A/V changes might give an early insight and a deeper understanding of the pathophysiology of such diseases. The A/V caliber ratio (Wong et al., 2004) has been used as a predictor for cardiovascular diseases. Current clinical methods for retinal vessel segmentation and A/V classification mainly rely on manual segmentation. However, due to the high complexity and diversity of vessel structures, manual segmentation brings inevitable shortcomings, including being time-consuming and laborious, having inter-rater variability and subjectivity, and having lower efficiency and accuracy. Thus, automatic methods for A/V classification and vessel segmentation are highly desirable in clinical settings. The advantages and disadvantages of current clinical methods and automatic methods are shown in Figure 1.

In recent years, several automated techniques have been proposed for retinal A/V classification (Ishikawa et al., 2005; Fraz et al., 2012a; Orlando et al., 2017). These techniques may be categorized into graph-based (Dashtbozorg et al., 2014; Joshi et al., 2014; Estrada et al., 2015; Hu et al., 2015; Pellegrini et al., 2018; Srinidhi et al., 2019) and feature-based (Niemeijer et al., 2009; Zamperini et al., 2012; Mirsharif et al., 2013; Xu et al., 2017; Huang et al., 2018a,b) techniques. Yet, in graph-based approaches, difficulties may be encountered when some vascular regions cannot be segmented, and hence, vessel segments cannot be reliably linked (Welikala et al., 2017). Besides, for feature-based techniques, most recent studies use a two-stage approach for retinal A/V classification. Vessels are firstly segmented from the background; next, the segmented vessels are categorized into arteries and veins by using purely handcrafted features in feature-based methods or by merging edge information in graph-based methods. However, the two-stage approach suffers from the heavy dependence of the A/V classification outcomes on vessel segmentation accuracy. In fact, if the accuracy of blood vessel segmentation is low in the first stage, the A/V classification results will not be good either in the second stage.

With the development of deep learning, many convolutional neural network-based methods have been proposed for joint vessel segmentation and A/V classification. Xu et al. (2018) adopted an improved fully convolutional network (FCN) architecture to segment retinal arteries and veins simultaneously. This method enabled end-to-end multilabel segmentation of color fundus images. AlBadawi and Fraz (2018) proposed an FCN architecture with an encoder–decoder structure for pixel-based A/V categorization. Meyer et al. (2018) also adopted the FCN architecture for A/V classification and demonstrated high performance on major vessels with thicknesses of more than three pixels. Hemelings et al. (2019) proposed a novel FCN-based U-Net architecture for simultaneous blood vessel semantic segmentation and A/V discrimination. Ma et al. (2019) proposed an enhanced deep architecture with a spatial activation mechanism for joint vessel segmentation and A/V identification. Li et al. (2020) made a highly confident prediction about the peripheral vessels by taking the structural information among vessels into account with post-processing.

**FIGURE 2:**
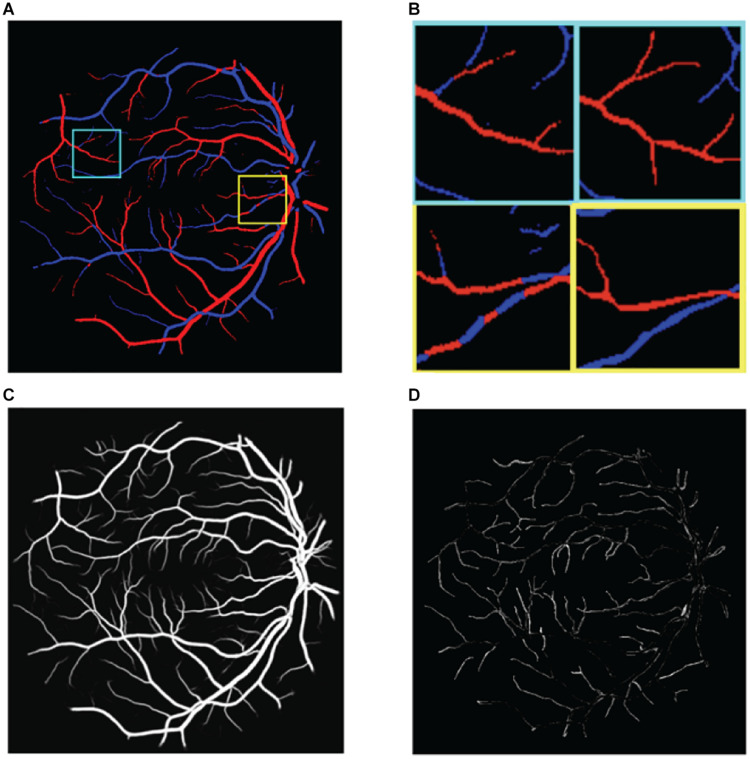
Illustration of the challenges in classifying retinal blood vessels. The results shown in the figure are from U-Net. **(A)** The results map of artery and vein, **(B)** two regions of interest in panel **(A)** are magnified. Left is prediction and right is ground truth, **(C)** the probabilities of vessel, and **(D)** the vessel errors compared with ground truth.

However, automatic vessel segmentation and A/V classification are still considered difficult tasks due to the following challenges:

(1) The multiscale structure of blood vessels is easily overlooked. These methods focus on large-scale structures such as thick blood vessels, but the performance is poor for small-scale structures such as the edge and the end of thick blood vessels, as shown in Figure 2D.

(2) There is extreme imbalance between positive samples (blood vessels) and negative samples (non-vessel areas) in retinal fundus images, where blood vessels account for only 15% of the whole image. Correspondingly, the proportion of arteries and veins is only about 7.5% each. As a result, directly classifying the pixels of the retinal image as background, artery, and vein pixels is very challenging.
(3) Distinguishing between arteries and veins can be highly confusing. The results of the aforementioned methods still show poor localization performance between arteries and veins; for example, the same blood vessel may be half recognized as an artery and half as a vein, as shown in Figure 2B.
(4) The choroid is similar to blood vessels and is easy to misclassify.

Besides, most of these existing methods are only validated on specific datasets. However, in clinical applications, the performance would underperform when tested on datasets with a different image resolution, imaging equipment, and population. For example, when generalizing a trained model to datasets with different center scales, the performance of the model usually deteriorates. The characteristic differences of retinal fundus images among different scales will also influence the segmentation results. One possible solution to this problem is labeling some samples of the new dataset to fine-tune the pre-trained model, but this process is expensive and time-consuming.

In order to alleviate these challenges, in this work, we introduce a novel convolutional neural network for joint A/V classification and vessel segmentation in retinal fundus images, named the vessel-constraint network (VC-Net). Firstly, in order to alleviate challenge (1), the VC-Net employs a vessel-constraint (VC) module to enhance the microvessels and the edge of thick vessels by using Gaussian kernel function probability maps to enhance the feature weights of the blood vessel edge area. And the multiscale feature (MSF) module is proposed to extract and express blood vessel features at different scales in the encoder. Secondly, in order to alleviate challenges (2)–(4), the VC module combines the global and local vessel information to generate a weight map to constrain the A/V features, which suppresses the background features. Not only can this alleviate the imbalance of positive and negative samples, but this also pays more attention to the features of arteries and veins to achieve better A/V classification performance.

The key contributions of this study can be highlighted as follows:

• For the first time, we propose a VC-Net that uses vessel probability information to constrain A/V and enhance learning of discriminative A/V features. In addition, the VC-Net can also get blood vessel segmentation results simultaneously.
• The newly designed VC module is powerful in A/V feature extraction. The VC module is used to capture the distribution information of vessels as a weight to constrain the A/V features, which suppresses background-prone features to pay more attention to vessel features. Data fusion (DF) alleviates well the problem of imbalance between positive and negative samples and helps us learn more discriminative A/V features. At the same time, the VC module enhances the microvessels and the edge of thick vessels by using Gaussian kernel function probability maps to improve the feature weights of the blood vessel edge area.
• The MSF module of multiscale DF is proposed to extract and express blood vessel features at different scales in the encoder, where the diameters of the main vessels and microvessels vary greatly. The DF training strategy is applied to improve the robustness of the model by fusing information from datasets with different scales.
• We publicly released the Tongren dataset with ground truth annotation. The lack of retinal fundus image data with annotated label impedes further exploration of retinal vessel-related researches such as vessel segmentation and A/V classification in the deep-learning community. Therefore, we established a dataset to promote these studies with a detailed data description in the experimental setup section of this paper.

The rest of this paper is organized as follows. Firstly, we present the details of our proposed methodology in Section “Materials and Methods”. Then, the descriptions of the datasets and the experimental details are described in Section “Experimental Setup”. Next, our experimental results are presented in Section “Results”. Finally, the discussions and conclusions follow in Section “Discussion and Conclusion.”

## Materials and Methods

**FIGURE 3:**
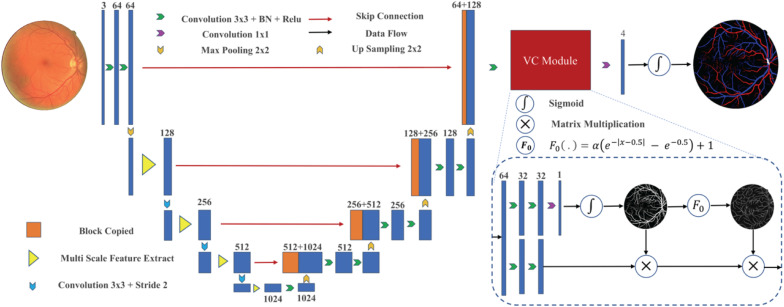
A block diagram of the proposed vessel-constraint network (VC-Net) architecture.

The design details of the VC-Net are shown in Figure 3. Firstly, we propose a VC module to capture the DF feature of the distribution and edge information of the vessel and enhance the microvessel and the edge of thick vessels. Then the distribution and enhanced information are utilized as weights to activate the A/V features and enforce the A/V classification module to focus more on vessels and help us learn more discriminative A/V features, for extremely unbalanced vessel and background. In addition, we used the MSF module to extract blood vessel features at different scales for varied diameters of the main vessels and microvessels.

### VC Module

In a retinal fundus image, blood vessels typically account for about 15% of the full image. Consequently, the area proportion of the arteries and veins is only about 7.5% each. Hence, directly classifying the retinal image pixels into background, artery, vein, and undecided pixels is a significant challenge task due to the high-class imbalance and the scarcity of training samples. To alleviate this problem, we designed a VC module at the end of the framework to enhance A/V classification.

The VC module combines the local and global vessel information to generate a weight map to constrain the A/V features, which suppresses the background-prone features to pay more attention to vessel features. In this way, it can alleviate the problem of severe imbalance between positive and negative samples. At the same time, we introduced Gaussian kernel function probability maps to improve feature weights of microvessels and the blood vessel edge area, thereby enhancing the feature representation of microvessels and the edge of thick vessels. The Gaussian activation function of the VC module is defined as

$$F\,\left(x\right)=\alpha \,\left(e^{-\left|x-0.5\right|}\,-\,e^{-0.5}\right)+1$$

Where *x* belongs to the probability map of the whole blood vessel segmentation, with values between 0 and 1, and α is a fixed parameter (set to 1 in this experiment).

The function *F*(*x*) further focuses on local vessel information, such as vascular boundaries and microvascular areas. Based on experimental observations and earlier studies, the probability of misclassifying vessel pixels is essentially concentrated around 0.5. These misclassified pixels come either from the vessel-background boundary or the microvascular areas whose features are not obvious and difficult to distinguish from the background. The background and thick vessel pixels have a value near 0 or 1.

Through the function *F*(*x*), the activation weight value of a pixel with a probability close to 0.5 was increased to [α(1 – *e*^–0.5^) + 1], while the activation weight values of the background and main thick vessels were set close to 1. The activation function constrains the activation weight value to be within [1, α(1 – *e*^–0.5^) + 1]. Note that *F*(0.5 + *x*_1_), *x*_1_ ∈ [0,0.5].

### Multiscale Feature

**FIGURE 4:**
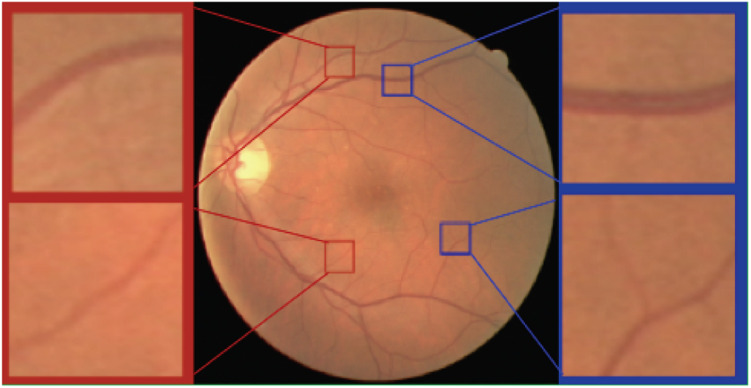
Arteries and veins of different scales in the retinal fundus images. *Top left* a major artery. *Top right* a major vein. *Bottom left* a minor artery. *Bottom right* a minor vein.

As shown in Figure 4, the scale of blood vessels varies greatly in retinal fundus images. On the one hand, the average artery diameter is generally slightly smaller than the average vein diameter. On the other hand, the average diameter for the main blood vessels is much larger than that of the capillaries.

**FIGURE 5:**
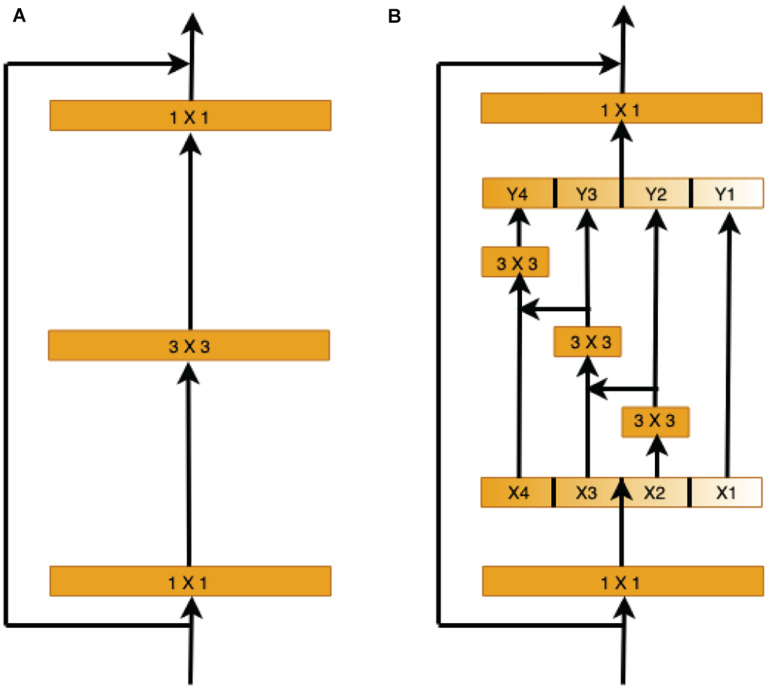
Comparison of the ResNet and Res2Net blocks (with a scale dimension of *k* = 4). **(A)** The conventional ResNet building block in CNN architectures. **(B)** The multi-scale feature (MSF) module of Res2Net uses a group of 3 × 3 filters.

Therefore, we use the capabilities of the pre-trained Res2Net (Gao et al., 2019) model to learn and understand the retinal vessel image features at different scales in the encoder stage. Instead of extracting features using 3 × 3 filter groups as in the ResNet (He et al., 2016) bottleneck block (Figure 5A), smaller filter groups connected in a hierarchical residual-type manner are used (Figure 5B). After the 1 × 1 convolutional stage, the features are split into *k* subsets, where the *i*th subset is denoted by *x*_*i*_, where *i* ∈ {1, 2,…, *k*}. While all subsets have the same spatial size, the channel count for each subset is 1/*k* times that of the input feature map. Each subset *x*_*i*_ (except for *x*_1_) has a 3 × 3 convolutional filter *F*_*i*_(). Thus, the filter output *y*_*i*_ can be written as

$$y_{i}=\left\{ \begin{array}{c} \,x_{i}\;i=1;\\ F_{i}\,\left(x_{i}\right)\;i=2;\\ F_{i}\,\left(\left(x_{i};\,y_{i-1}\,\right)\right)\,2<i\leq k. \end{array}\right.$$

Each 3 × 3 convolutional operator *F*_*i*_() might get information from all feature subsets {*x*_*j*_, *j* ≤ *i*}. When a feature subset *x*_*j*_ is processed by a 3 × 3 convolutional operator, the output result may have an enlarged receptive field compared to *x*_*j*_.

Here, the scale dimension *k* is used as a control parameter. A larger *k* value enables learning features with larger receptive field sizes, with insignificant computation and memory overheads due to concatenation.

### Loss Function

We employ an end-to-end deep-learning scheme as our underlying framework. The A/V loss is quantified by the commonly used cross-entropy loss function

$$L\,\_\,A\,V_{c\,e}=-\frac{1}{n}\,\sum_{i\,=1}^{n}\left(y_{i}\,\log\left(y_{i}^{{}^{\prime }}\right)+\left(1\,-\,y_{i}\right)\,\text{log}\,\left(1-\,y_{i}^{{}^{\prime }}\right)\right)$$

While the vessel segmentation loss is quantified by the binary cross-entropy

$$L\,\_\,V_{b\,c\,e}=-\frac{1}{n}\,\sum_{c=0}^{1}\sum_{i\,=1}^{n}y_{i}\,\log\left(y_{i}^{{}^{\prime }}\right)$$

Where *n* denotes the number of pixels in the input image, *y*’ is the predicted output probability of a foreground pixel, *y* is the ground-truth pixel label, and *c* denotes the *c*th class of the output. The total loss is defined as

$$\text{Loss}=\gamma *L\,\_\,A\,V_{c\,e}+\delta *L\,\_\,V_{b\,c\,e}+\beta *{||W||}_{2}^{2}$$

Where γ = 0.6, δ = 0.4, and δ + γ = 1. We use *L*_2_ regularization with a weight of β = 0.0002.

## Experimental Setup

In this section, we describe the used retinal image datasets, the evaluation metrics for retinal vessel segmentation and A/V classification, and the VC-Net training details.

### Datasets

In this work, we evaluated our approach and assessed its clinical applicability on five retinal fundus image datasets of different scales. Three datasets are publicly available while the other two were collected by authors. In order to validate the generalization performance and robustness of our method by DF experiments, we specifically annotated two multiscale datasets. An overview of these datasets is given in Table 1.

**TABLE 1:** Overview of datasets used for artery/vein (A/V) classification and vessel segmentation.

Datasets	# images	Resolution
DRIVE (Hu et al., 2013)	40	584 × 565
LES (Orlando et al., 2018)	22	1,444 × 1,620, 1,958 × 2,196
HRF (Odstrcilik et al., 2013)	45	3,304 × 2,336
Tongren	30	1,888 × 2,816
Kailuan	30	(1,588–2,112) × (1,586–2,112)

#### DRIVE

Our model was firstly trained and tested on the publicly available DRIVE database (Hu et al., 2013). This database contains 40 color retinal fundus images with image dimensions of 584 × 565 pixels. These images were evenly divided into training and test sets with 20 images in each set. Pixel-wise labeling is provided for vessel segmentation and A/V classification.

#### LES

The LES dataset (Orlando et al., 2018) contains 22 images with a 30° field of view (FOV) and a resolution of 1,444 × 1,620 pixels for 21 images and a 45° FOV and a resolution of 1,958 × 2,196 pixels for one image. The images are equally divided into training and test sets with 11 images in each set.

#### HRF

The HRF dataset (Odstrcilik et al., 2013) contains 45 images equally divided among three categories, namely, healthy subjects, patients with diabetic retinopathy, and patients with glaucoma. Images were captured with an FOV of 60° and a pixel resolution of 3,304 × 2,336. Only one ground-truth segmentation map is available for each image. For each category, five images are used for training and the rest are used for testing.

#### Tongren

The Tongren clinical dataset contains 30 representative retinal fundus images with a 45° FOV and a resolution of 1,888 × 2,816 pixels, within which 20 images were normal and 10 images were of moderate cataract or retinal diseases including glaucoma, age-related macular degeneration, and retinal vein occlusion. An approval was obtained from the Ethics Committee of Beijing Tongren Hospital. The ocular fundus had been taken with a fundus camera (CR6-45NM camera, Canon Inc., Ota, Tokyo, Japan). These images were labeled by two experienced ophthalmologists with the ITK-SNAP toolkit (Yushkevich et al., 2006). For each category, half of the images are used for training, and the rest are used for testing.

#### Kailuan

The Kailuan database contains 30 images which were collected from participants of the community-based Kailuan Cohort Study (Jiang et al., 2015). These images have different sizes. The minimum, average, and maximum heights are 1,588, 1,902, and 2,112. The minimum, average, and maximum widths are 1,586, 1,901, and 2,112. We used 15 images for training and the rest for testing. Also, these images were labeled by experienced ophthalmologists with the ITK-SNAP toolkit (Yushkevich et al., 2006).

The binary ground-truth segmentation maps for the DRIVE, LES, and HRF images are publicly available. For the Tongren and Kailuan images, we have manually created FOV masks using methods similar to those of Soares et al. (2006), Figure 6 shows samples of Tongren and Kailuan datasets.

**FIGURE 6:**
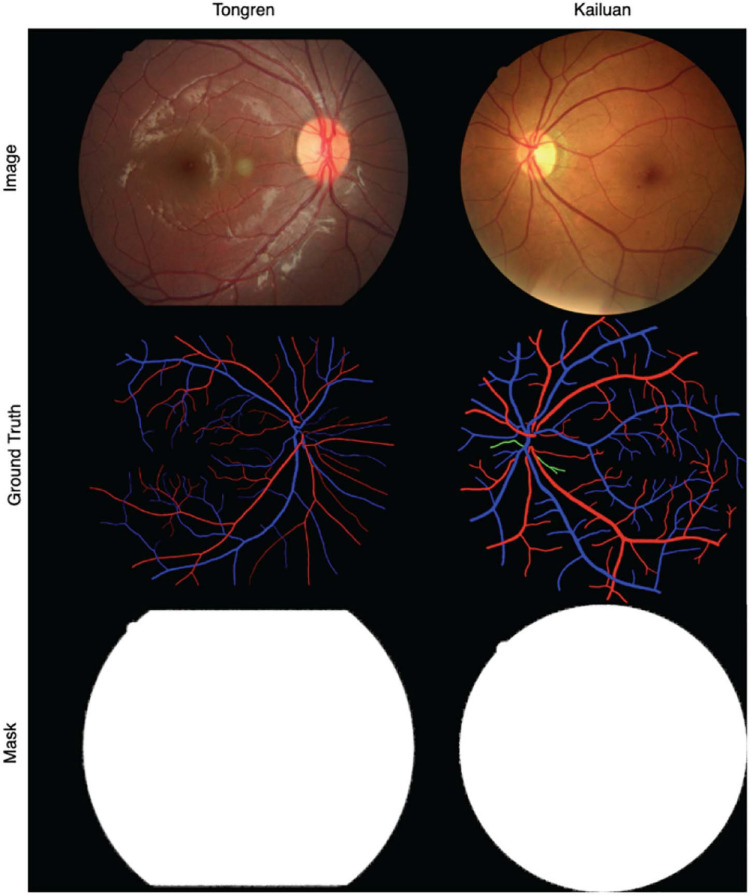
Sample images and vessel visualization maps for the Tongren (*left*) and Kailuan (*right*) image databases.

### Evaluation Metrics

The retinal vessel segmentation outcomes of the proposed method were compared against those of other reference methods using several metrics, namely, sensitivity (SE), specificity (SP), accuracy (ACC), the area under the ROC curve (AUC), and the F1 score (F1). The binary segmentation maps were generated through thresholding the probability maps with a 0.5 threshold.

For A/V classification, five performance evaluation metrics were adopted. We interpret arteries as positives and veins as negatives. The A/V sensitivity (SE_*AV*_) and specificity (SP_*AV*_) reflect the model capability for correctly detecting arteries and veins, respectively. The balance accuracy (BACC) quantifies the overall performance of the model. These metrics are defined as follows.

$${\text{SE}}_{\text{AV}}=\frac{\text{TP}}{\text{TP}+\text{FN}}$$

$${\text{SP}}_{\text{AV}}=\frac{\text{TN}}{\text{TN}+\text{FP}}$$

$$\text{BACC}=\frac{{\text{SE}}_{\text{AV}}+{\text{SP}}_{\text{AV}}}{2}$$

Where TP is the count of the correctly classified artery pixels, TN is the count of the correctly classified vein pixels, FP is the count of the vein pixels misclassified as artery pixels, and FN is the count of the artery pixels misclassified as vein pixels.

In addition, we compute the F1 score for arteries (F1_*A*_) and the F1 score for veins (F1_*V*_) when arteries and veins represent the relevant samples, respectively. The optimal value for each of these metrics is 1. Computations were restricted to pixels within the FOV.

### Network Training Details

Few training samples are available in each of the five databases and are hence insufficient for handling model complexity. To alleviate this problem, several data augmentation strategies (Fraz et al., 2012b; Maninis et al., 2016; Feng et al., 2017; Oliveira et al., 2018; Guo et al., 2019) have been explored, including image scaling with different scale factors and image rotation by different angels. As no prior knowledge is available on the appropriate patch size selection, patches with a size of 512 × 512 were randomly picked from the retinal images and used for network training. For each test image, ordered patches were collected, and the final segmentation and classification outcomes were found by stitching together the associated patch predictions. A stochastic gradient descent algorithm with momentum was employed for optimizing model parameters with a maximum of 4,000 iterations. The learning rate was initially set to 0.001 and then cut in half every 1,500 iterations. Method implementations were carried out using a PyTorch backend the NVIDIA CUDA^®^ Deep Neural Network library (cuDNN 9.0), and an Intel^®^ Xeon^®^ Gold 6148 CPU with a processor of 2.40 GHz, a RAM of 256 GB, and an Ubuntu 16.04 operating system.

## Results

In this section, we introduce the results of the experiment. Firstly, we conduct a series of ablation studies to systematically analyze the effectiveness of each component of the proposed network and its impacts on overall segmentation performance. Then, we apply our method to the aforementioned datasets and compare it with state-of-the-art methods. Finally, we verify the effectiveness of the DF strategy to address the challenges in new datasets.

### Ablation Studies

Detailed ablation studies have been conducted to evaluate the contribution of each module of the proposed VC-Net architecture. These modules include the basic U-Net module, the MSF in the encoder, and the VC module for A/V classification. In Table 2, the first two methods apply direct recognition of retinal fundus images into background, artery, vein, and undecided pixels. Based on the recognition results, vessel segmentation indicators are calculated. The proposed method was used for vessel segmentation and A/V classification simultaneously; performance indices were calculated accordingly.

**TABLE 2:** Results of the ablation study for A/V classification (α = 1.0).

Methods			A/V classification				
	
U-Net	MSF	VC	BACC	SE_*AV*_	SP_*AV*_	F1_*A*_	F1_*V*_
✓	×	×	0.9118	0.8950	0.9287	0.7089	0.7586
✓	✓	×	0.9481	0.9251	0.9711	0.7433	0.7861
✓	×	✓	0.9483	0.9327	0.9547	0.7428	0.7880
✓	✓	✓	**0.9542**	**0.9351**	**0.9732**	**0.7605**	**0.7971**

As shown in Table 2, the A/V classification results have been significantly improved with the addition of MSF. The MSF can extract and express the vessel features with different scales in the encoder to solve the varying diameters of the main vessels and microvessels. Remarkably, the blood vessel classification performance has been further improved to a certain extent by using the VC module; our results show that we achieved 0.9483, 0.9327, 0.9547, 0.7428, and 0.7880 on BACC, SE_*AV*_, SP_*AV*_, F1_*A*_, and F1_*V*_, respectively. The VC module can suppress background-prone features to pay more attention to vessel features; it alleviates well the problem of positive and negative sample imbalance and helps us learn more discriminative A/V features. At the same time, the VC module can enhance the feature representation of microvessels and the edge of thick vessels. More importantly, from Table 2, we can see that the combination of U-Net, MSF, and VC modules achieves the best results with a BACC of 0.9542, SE_*AV*_ of 0.9351, SP_*AV*_ of 0.9732, F1_*A*_ of 0.7605, and F1_*V*_ of 0.7971. Therefore, the ablation study demonstrates the effectiveness of the proposed modules.

**FIGURE 7:**
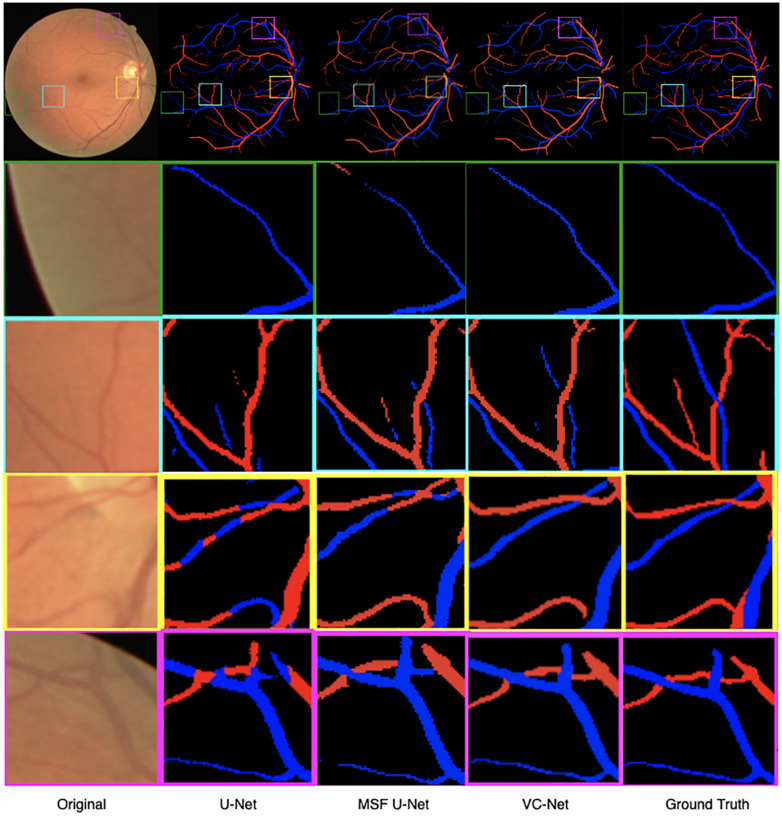
Retinal fundus images and vessel maps for different modules. Four regions of interest are highlighted and magnified in rows 2–5.

As shown in Figure 7, we visualized the A/V classification results for different modules of the proposed VC-Net architecture. In particular, results for four regions of interest were highlighted and magnified. We can see that A/V classification results of the U-Net are poor, where arteries and veins are seriously confused, and that there are many misclassifications at the edges and ends of blood vessels. With the introduction of MSF, the A/V classification results have been improved, but there is still the problem of arteries and veins being confused near the crossing and branching points of blood vessels. Obviously, in comparison with other models, we proposed the VC-Net as it achieves better A/V classification results both locally and globally. The above analysis proves that our model certainly improves the overall A/V classification performance.

As we can see, the VC-Net model outperformed other methods based on performance metrics and visualization results. In addition, we also explored the influence of varying the α parameter values on the VC-Net model performance. Specifically, we trained the model from scratch with different α values, ranging from 0.4 to 1.6. The results are shown in Table 3. For A/V classification, the BACC, SE_*AV*_, F1_*A*_, and F1_*V*_ metrics increased with the decrease in α. Nevertheless, the increase between α = 0.4 and α = 0.6 was very small, and there was even a small decrease in F1_*A*_ and F1_*V*_. For vessel segmentation, α approaches 1.0–1.4, and the indicators show good performance. Therefore, the α value should be adjusted according to different scenarios. If a larger SE_*AV*_ value is desired, the α value can be appropriately reduced to train a model from scratch.

**TABLE 3:** The effect of α on vessel segmentation and classification training (VC-Net model training from scratch).

α	Segmentations					A/V classification				
		
	ACC	SE	SP	AUC	F1	BACC	SE_*AV*_	SP_*AV*_	F1_*A*_	F1_*V*_
0.4	0.9566	0.8302	0.9755	0.9799	0.8290	**0.9570**	**0.9405**	0.9735	0.7633	0.7988
0.6	0.9565	0.8311	0.9752	0.9801	0.8287	0.9568	0.9397	0.9740	**0.7634**	**0.7989**
0.8	0.9565	0.8305	0.9753	0.9803	0.8286	0.9563	0.9385	0.9740	0.7622	0.7985
1.0	**0.9570**	0.8258	**0.9766**	0.9812	**0.8296**	0.9542	0.9351	0.9732	0.7605	0.7971
1.2	0.9568	0.8288	0.9759	0.9804	0.8294	0.9535	0.9357	0.9714	0.7616	0.7954
1.4	0.9557	**0.8475**	0.9720	**0.9814**	0.8290	0.9540	0.9352	0.9728	0.7607	0.7963
1.6	0.9565	0.8261	0.9763	0.9811	0.8289	0.9564	0.9354	**0.9773**	0.7595	0.7963

After training the VC-Net model, we varied the α values in the trained model to test the model performance on the test dataset. The results are shown in Table 4. Obviously, with the decrease of α, most indicators are increased except SE, F1, and F1_*V*_. As a bonus and as SE is increased with α, the effectiveness of the VC model is verified from the side. When a model has been trained, if a larger indicator for A/V classification is needed, α can be appropriately reduced. And if a lager SE is needed, α can be appropriately increased.

**TABLE 4:** The effect of α on vessel segmentation and classification testing (the VC-Net model has been trained).

α	Segmentations				A/V classification				
		
	ACC	SE	SP	F1	BACC	SE_*AV*_	SP_*AV*_	F1_*A*_	F1_*V*_
0.4	0.9574	0.7848	**0.9830**	0.8236	**0.9554**	**0.9360**	**0.9748**	**0.7616**	0.7964
0.6	**0.9575**	0.8015	0.9807	0.8270	0.9549	0.9356	0.9742	0.7615	0.7968
0.8	0.9573	0.8148	0.9786	0.8287	0.9545	0.9354	0.9737	0.7612	0.7970
1.0	0.9570	0.8258	0.9766	0.8296	0.9542	0.9351	0.9732	0.7605	0.7971
1.2	0.9566	0.8351	0.9748	**0.8299**	0.9539	0.9349	0.9729	0.7599	**0.7973**
1.4	0.9562	0.8429	0.9732	0.8298	0.9536	0.9348	0.9725	0.7587	0.7970
1.6	0.9557	**0.8496**	0.9716	0.8293	0.9535	0.9346	0.9723	0.7573	0.7966

### Comparison With Existing Methods on the DRIVE Dataset

We compared the VC-Net performance with that of other state-of-the-art methods on the DRIVE dataset for vessel segmentation and A/V classification tasks. Table 5 summarizes the vessel segmentation comparison results. As seen, the proposed VC-Net shows superior segmentation performance in terms of AUC and F1. In Table 6, the existing methods are evaluated for the classification performance on the segmented vessels only. On the contrary, we evaluated the VC-Net performance on all A/V ground-truth pixels. This evaluation is more challenging than that on the segmented vessels, since the identification of major vessels is an easier task if the capillary vessels are not segmented. The comparison with existing methods under the same criteria shows superior performance of our model, which achieves a BACC of 0.9554, SE_*AV*_ of 0.9360, SP_*AV*_ of 0.9748, F1_*A*_ of 0.7616, and F1_*V*_ of 0.7964. Indeed, our model surpasses the current best A/V classification method due to the introduction of the VC module.

**TABLE 5:** Vessel segmentation results of vessel-constraint network (VC-Net) and other existing methods on the DRIVE dataset.

Methods	ACC	SE	SP	AUC	F1
U-Net (Ronneberger et al., 2015)	0.9541	**0.8319**	0.9713	0.9750	0.8162
DDNet (Mou et al., 2019a)	0.9594	0.8126	0.9788	0.9796	N/A
AC_Net (Ma et al., 2019)	0.9570	0.7916	0.9811	0.9810	N/A
CS-Net (Mou et al., 2019b)	**0.9632**	0.8170	**0.9854**	0.9798	N/A
CE-Net (Gu et al., 2019)	0.9545	0.8309	0.9747	0.9779	N/A
RU-Net (Alom et al., 2018)	0.9556	0.7792	0.9813	0.9784	0.8171
BTS-UNet (Guo et al., 2019)	0.9551	0.7800	0.9806	0.9796	0.8208
DE-UNet (Wang et al., 2019)	0.9567	0.7940	0.9816	0.9772	0.8270
**VC-Net (α = 1)**	0.9570	0.8258	0.9766	**0.9812**	**0.8296**

*N/A, not available.*

**TABLE 6:** Artery/vein (A/V) classification results of VC-Net and other existing methods on the DRIVE dataset.

Methods	BACC	SE_*AV*_	SP_*AV*_	F1_*A*_	F1_*V*_
Dashtbozorg et al., 2014	0.8740	0.9000	0.8400	N/A	N/A
Estrada et al., 2015	0.9350	0.9300	0.9410	N/A	N/A
U-Net (Ronneberger et al., 2015)	0.9122	0.9145	0.9083	0.7089	0.7586
Xu et al., 2017	0.9230	0.9290	0.9150	N/A	N/A
DOS (Zhao et al., 2018)	N/A	0.9190	0.9150	N/A	N/A
AC_Net (Ma et al., 2019)	0.9450	0.9340	0.9550	N/A	N/A
VC-Net (α = 1)	0.9542	0.9351	0.9732	0.7605	**0.7971**
**VC-Net (α = 0.4)**	**0.9554**	**0.9360**	**0.9748**	**0.7616**	0.7964

In particular, for Table 6, it is noteworthy that VC-Net has outperformed existing methods in terms of all metrics in identifying arteries and veins. This performance superiority is mainly due to the fact that the vessel activation map not only enhanced the vascular boundaries and microvessels but also strengthened the main thick vessels, suppressed the background, and hence enabled the model to learn more vessel features. Besides, the vessel activation map eliminated the imbalance between the background and the blood vessel samples to a certain extent.

### Comparison With Existing Methods on Other Datasets

The proposed VC-Net was also compared with existing methods on two other public datasets and two collected datasets. For vessel segmentation, the results on the LES and HRF public datasets are shown in Table 7. Clearly, VC-Net achieved significantly better results with an ACC of 0.9663, SP of 0.9843, AUC of 0.9806, and F1 of 0.8101 in the HRF-AV dataset.

**TABLE 7:** Performance comparison for different vessel segmentation methods on the LES and HRF datasets.

Datasets	Methods	Vessel segmentation				
		
		ACC	SE	SP	AUC	F1
LES	FC-CRF (Orlando et al., 2017)	N/A	0.7874	0.9584	0.9359	0.7158
	Jloss (Yan et al., 2018)	0.9400	0.7900	0.9600	N/A	N/A
	**VC-Net (α = 1)**	**0.9722**	**0.8504**	**0.9840**	**0.9821**	**0.8417**
HRF	DNN (Samuel and Veeramalai, 2019)	0.8531	**0.8655**	0.8523	0.9665	N/A
	UA_VA (Galdran et al., 2019)	0.9100	0.8500	0.9100	0.9400	0.6200
	MF-Net (Odstrcilik et al., 2013)	0.9494	0.7741	0.9669	0.9670	0.7316
	FCN-TL (Jiang et al., 2018)	0.9662	0.7686	0.9826	0.9770	N/A
	**VC-Net (α = 1)**	**0.9663**	0.7903	**0.9843**	**0.9806**	**0.8101**

The A/V classification outcomes are shown in Table 8. It can be seen that all indicators have been significantly improved compared to those in UA_VA (Galdran et al., 2019) on the LES dataset. In particular, the BACC, SE_*AV*_, and SP_*AV*_ metrics increased by 9.84, 7.10, and 11.38%, respectively. Moreover, the VC-Net also showed excellent performance on the HRF dataset with a BACC of 0.9646. The above results once again demonstrate the excellent performance of VC-Net.

**TABLE 8:** Performance comparison of different A/V classification methods on the LES and HRF datasets.

Datasets	Methods	A/V classification				
		
		BACC	SE_*AV*_	SP_*AV*_	F1_*A*_	F1_*V*_
LES	UA_VA (Galdran et al., 2019)	0.8600	0.8800	0.8500	N/A	N/A
	**VC-Net (α = 1)**	**0.9446**	**0.9425**	**0.9467**	**0.7635**	**0.7988**
HRF	**VC-Net (α = 1)**	**0.9646**	**0.9588**	**0.9704**	**0.7389**	**0.7839**

In addition, we tested the VC-Net performance for blood vessel segmentation and A/V classification on the two collected Tongren and Kailuan datasets. The results are shown in Table 9. For the Tongren dataset, there were significant improvements compared with the previous methods. Specifically, the ACC, BACC, F1_*A*_, and F1_*V*_ metrics for VC-Net were improved by 0.39, 4.41, 6.43, and 7.44%, respectively, in comparison with the basic U-Net method. And the VC-Net achieved better results with an SP of 0.9767, F1 of 0.7974, F1_*A*_ of 0.7221, and F1_*V*_ of 0.7741 on the Kailuan dataset. The experimental results demonstrate that our method achieves competitive performance for A/V classification and vessel segmentation.

**TABLE 9:** Vessel segmentation and A/V classification performance of different methods on the Tongren and Kailuan datasets (α = 1).

Datasets	Methods	Segmentations					A/V classification				
			
		ACC	SE	SP	AUC	F1	BACC	SE_*AV*_	SP_*AV*_	F1_*A*_	F1_*V*_
Tongren	U-Net (Ronneberger et al., 2015)	0.9637	**0.8283**	0.9752	0.9813	0.7798	0.9068	0.9138	0.9018	0.6903	0.7208
	S-UNet (Hu et al., 2019)	0.9652	0.7822	0.9830	**0.9824**	0.7994	N/A	N/A	N/A	N/A	N/A
	VC-Net	**0.9675**	0.7705	**0.9863**	0.9819	**0.8048**	**0.9468**	**0.9421**	**0.9516**	**0.7347**	**0.7744**
Kailuan	VC-Net	**0.9516**	**0.7961**	**0.9767**	**0.9766**	**0.7974**	**0.9442**	**0.9413**	**0.9472**	**0.7221**	**0.7741**

### Segmentation Results of Challenging Images

**FIGURE 8:**
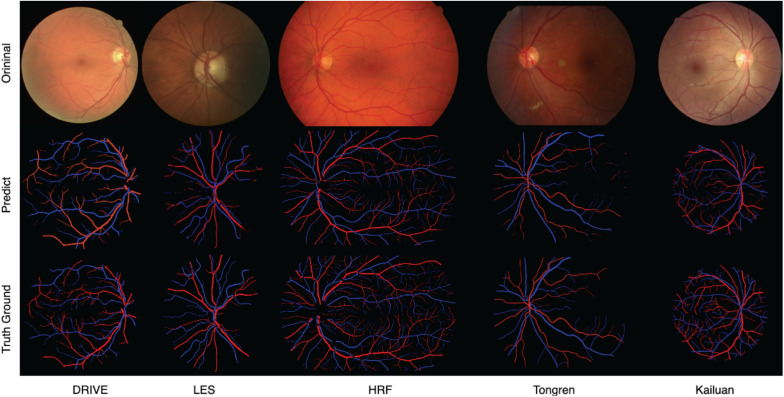
Predicted and ground-truth vessel maps for sample images from five retinal fundus image databases.

Sample images from the above-mentioned five databases and the corresponding predicted and ground-truth vessel maps are shown in Figure 8. Accurate segmentation of challenging images proves the effectiveness of our method. For the DRIVE and HRF datasets, both the overall and local vessel segmentation and A/V classification results are excellent with considerable continuity. Good results were achieved also on the other datasets, although the local results are not as well as those of the DRIVE and HRF datasets. Due to computational limitations, only patch-level networks can be trained on large-scale datasets, and hence, the results can be inferior to whole-image networks.

### Evaluation Results on Unseen Datasets With Multiscale DF

Data fusion is a fundamental step to deal with the new data problem. To improve the robustness of the proposed model, DF from datasets with different scales could enrich the amount of training data and data distribution and could be validated on a new dataset with multiscale. We define this training strategy as DF training.

The first three rows of Table 10 were only trained on the DRIVE, LES, or HRF datasets and tested on the Kailuan dataset. Finally, the three datasets combined and shuffled the images. The fused data are used as the training dataset and tested under the Kailuan dataset. It can be seen that the best results have been achieved on most indicators on the Kailuan dataset after DF. As a bonus, the DF training can enhance the robustness of the model, and it is more suitable for testing on new datasets.

**TABLE 10:** The model is trained under the selected training dataset and tested under the Kailuan dataset with multiscale.

Training datasets			A/V classification				
	
DRIVE	LES	HRF	BACC	SE_*AV*_	SP_*AV*_	F1_*A*_	F1_*V*_
✓	×	×	0.6086	0.4721	0.7451	0.2052	0.2870
×	✓	×	0.8776	0.8942	0.8610	0.6273	0.6803
×	×	✓	0.9251	0.8876	**0.9626**	0.6504	0.7375
✓	✓	✓	**0.9412**	**0.9297**	0.9562	**0.6790**	**0.7449**

## Discussion and Conclusion

In this paper, we propose a VC network that utilizes information of vessel distribution and edge to enhance A/V classification. The proposed VC module combines local and global vessel information to generate a more reasonable weight map to constrain the A/V features, which suppresses the background-prone features and enhances the edge and end features of blood vessels. Meanwhile, we used an MSF module to obtain multiscale vessel features, such as the main thick vessels, vascular boundaries, and microvascular regions. Our method achieves better blood vessel segmentation and A/V classification performance. More importantly, we adopt the DF strategy to improve the robustness and generalization ability of the proposed model.

The VC-Net model demonstrates the effectiveness on multiscale and multicenter datasets. It outperforms existing methods and achieves state-of-the-art results for A/V classification and vessel segmentation on three public datasets. And the proposed model was tested on multicenter datasets: Tongren and Kailuan; the results indicate the superior generalization capability of the network. In addition, this model shows better performance on datasets with different resolutions. The visualized vessel maps reflect the importance of the MSF extraction module in our model and the excellent overall control of the global and detailed features by the VC module. In particular, to promote the development of this field, we collected two retinal fundus image datasets (Tongren and Kailuan), which labeled the arteries and veins with the ITK-SNAP toolkit, and we will be releasing the Tongren dataset.

One of the limitations of our work is that large-scale fundus images cannot be accommodated by the network; such images should be reduced to patches of a reasonable size to facilitate the training and testing processes. This patch-based approach distorts the global view of capillaries and large vessels. The other limitation is that computational resources are highly demanding. Therefore, we hope to use our work as a basis to further analyze the performance of vessel segmentation and A/V classification algorithms for large-scale fundus images and improve the utilization of computational resources.

In the future, we will deploy our algorithm to mobile terminals and develop an automatic retinal blood vessel analysis system, which is more conducive to clinicians’ understanding and use of this algorithm and promotes the diagnosis of ophthalmology and systemic diseases.

## Data Availability Statement

The original contributions presented in the study are included in the article/supplementary material, further inquiries can be directed to the corresponding author/s.

## Author Contributions

JH: formal analysis, investigation, methodology, software, validation, and writing–original draft. HW: investigation, software, validation, and writing–original draft. ZC and GW: investigation and revise manuscript. JJ: supervision. YW: resources and supervision. JZ: funding acquisition, project administration, resources, and supervision. All authors contributed to the article and approved the submitted version.

## Conflict of Interest

The authors declare that the research was conducted in the absence of any commercial or financial relationships that could be construed as a potential conflict of interest.
